# Developing and Applying an Urban Resilience Index for the Evaluation of Declining Areas: A Case Study of South Korea’s Urban Regeneration Sites

**DOI:** 10.3390/ijerph20043653

**Published:** 2023-02-18

**Authors:** Byungsuk Kim, Gil-Sang Lee, Minjun Kim, Who-Seung Lee, Hee-Sun Choi

**Affiliations:** 1 Water and Land Research Group, Division for Environmental Planning, Korea Environment Institute, Sejong 30147, Republic of Korea; 2 Environmental Assessment Group, Division for Land Policy Assessment, Korea Environment Institute, Sejong 30147, Republic of Korea; 3 Department of Planning and Strategy, Korea Environment Institute, Sejong 30147, Republic of Korea

**Keywords:** urban regeneration, urban decline, urban resilience, urban disaster

## Abstract

This study attempts to identify the direction of urban regeneration projects in declining areas by using the concept of urban resilience to cope with climate change and disaster. To this end, urban resilience was classified into a Green Resilient Infrastructure (GRI) and an Interactive Safety System (ISS), through a review of previous studies, and categorized into vulnerability, adaptability, and transformability. A total of 12 detailed indicators were derived and indexed using Euclidean distance. Using the indicators, three Korean urban regeneration targets, in Daegu, Mokpo, and Seosan, were selected to evaluate resilience before and after the urban regeneration plan. Consequently, the postplanning resilience index improved in all three target sites, compared to before the regeneration plan. Additionally, previously the regeneration plan showed lower index values in comparison to places not designated as urban regeneration areas. These results suggest that urban resilience needs to be considered in future urban regeneration projects, and that resilience indicators can be used as a means to set the direction of urban regeneration projects. To improve the overall resilience of a region, these indices can help local government establish a reference point for urban resilience in its region.

## 1. Introduction

Resilience has been a well-known concept in engineering and ecology, and recently, interest in “regional resilience” has emerged in various fields, including social science and regional planning [1]. It is used in fields such as psychology, pathology, economics, education, material engineering, and urban planning. In addition to the increase in resilience research in urban planning, there are more studies related to resilience in other fields amid rising economic and social vulnerability [2,3,4,5,6]. According to research [5], diverse terms related to resilience have been mentioned in previous studies, such as “resilience”, “flexibility”, and “recovery”, and can be applied at various levels, from global to country, region, city, community, and individual levels. The concept of resilience can also be considered in various fields, including climate change or disasters, such as flood or fire.

Currently, the impact and damage caused by climate change and disasters are increasing worldwide, and efforts are being made to secure a foundation to respond to external shocks at the national, urban, and community levels [3]—Korea is no exception. In Korea, damage caused by disasters such as heavy rain, heat waves, strong winds, and local floods have continued to occur; the frequency of natural disasters and the magnitude of damage have been steadily increasing [6]. In particular, urban regeneration areas with poor physical and social conditions are highly likely to cause disasters because of the concentration of old and weak houses, a lack of urban infrastructure, poor management, and the concentration of vulnerable people [7]. Amid these changes, the regional disaster prevention paradigm is shifting toward “resilience” [8], and the focus is on improving sustainability by strengthening resilience in disaster-vulnerable urban regeneration areas.

In terms of disaster, resilience is a concept that encompasses the ability to prevent, prepare, respond to, and recover from a disaster. It is crucial for the quality of life of local residents and the safety of residents in urban regeneration areas [6]. Therefore, it is necessary to understand the physical, social, and environmental status, and the characteristics of the region, to prepare tailored countermeasures for the region against disasters. This study aims to select a quantitative evaluation index that can assess the level of resilience in urban regeneration areas. Additionally, it is intended to seek the direction of urban regeneration projects to secure urban resilience through the evaluation of urban regeneration areas, using the selected indicators.

## 2. Background

### 2.1. Urban Decline and Regeneration

In South Korea, population concentration in cities occurred because of rapid urbanization and industrialization—presently, this situation has become serious. The population of the Seoul metropolitan area, which includes Seoul, Gyeonggi, and Incheon, stood at 26.04 million as of July 2022, with more than 50% of the total population living in the metropolitan area. Such social phenomena have caused problems in declining cities, including the aging of the residential environment, population decline, population concentration in metropolitan areas, and changes in industrial structure. The Korean government tried to solve these urban problems through urban regeneration projects. Urban regeneration refers to revitalizing declining cities economically, socially, physically, and environmentally, by strengthening regional capabilities, introducing and creating new functions, and utilizing local resources [9].

The three indicators of urban decline in Korea are a “decrease in the number of businesses”, a “decrease in population”, and the “deterioration of the living environment” [9]. Urban decline and regeneration issues are emerging, and related studies are underway in various fields. In terms of the decline of industry and the decrease of jobs, which cause urban decline, Han and Nam [10] show a significant relationship between the decline of industrial complexes in urban areas, and urban decline. Residents’ satisfaction may decrease in declining areas because of job losses and the departure of residents. However, in the case of residential satisfaction, it may vary, depending on the conditions and circumstances of the declining city. In large cities, satisfaction with the residential environment decreases because of the decline, but overall life satisfaction increases as social costs decrease, because of relief from overcrowding. Nevertheless, in small cities, the decline of the city decreases overall life satisfaction, family income satisfaction, and residential environment satisfaction [11].

Population outflow can eventually result in the destruction of communities in a region. Communities are the driving force of local production, consumption, and culture, and play a significant role in enhancing sustainability and promoting self-sustainability. Additionally, strategies to ensure regional sustainability through community revitalization become a source of regional social resilience [12]. Social resilience refers to the ability of the human community to withstand external shocks, such as politics, economy, society, and the environment [13]. This may be why central and local governments value community recovery in urban regeneration projects. In this context, one of the concepts that account for a large part of urban regeneration research is “resident participation”; various studies [14,15,16,17,18] have investigated this phenomenon. In other words, community-based resident participation is key to the success of urban regeneration.

The decrease in jobs and population naturally accelerates the aging of the area, leading to social problems such as vacant houses, crime, worsening living conditions, and vulnerability to disasters. Among these, vacant houses represent one of the most pressing problems in declining areas. Therefore, it is necessary to expand the national budget for vacant-house management through the analysis of the distribution of vacant houses for the systematic management of urban decay areas and to establish a comprehensive management system to provide vacant-house surveys and DBs [19]. The aging of the living environment and community collapse may worsen damage in the case of disasters in declining areas and add difficulties in recovery. In the study of Lim et al. [20], eight indices related to disasters were developed. The study showed that risk is more severe in areas that were more declined. Additionally, various studies related to urban decline and disaster risk have been conducted, including a study on future climate change vulnerability-evaluation methodology [21]. Such work supports preventive disaster response and provides vulnerability information on heavy snow, the ratio of children under 15 years and people aged 65 years or older, and the ratio of old buildings [22].

### 2.2. Urban Resilience

Different international organizations, such as the Organization for Economic Co-operation and Development (OECD), UN-HABITAT, Resilient Europe, and Global Alliance for Resilience, have defined urban resilience differently (Table 1). However, the definitions mostly refer to resistance, absorption, adaptation, transformation, change, recovery, and the preparation of a city, community, home, organization, or company, against events (shock, stress, risk, disaster, etc.,) or possible risk [23].

Overall, the resilience of cities is defined as: “a concept that does not simply mean returning to its original state but includes a new level of transformability at some point while securing stability in pre-crisis states and adaptability to the changed environment” [24,25,26]. Risk indicators for declining areas can be confused sometimes with the concept of resilience indicators; however, the two indicators are different. The difference between the resilience index compared to the existing risk index is that it includes the potential for development after external shocks. Additionally, as urban resilience considers the potential of a region, it may be a significant factor in meeting the purpose of urban regeneration to revitalize a declining region.

In this study, urban resilience is defined as the potential and various response capabilities of a city against disasters that may occur, based on previous studies. The ability to prevent the occurrence of a disaster, minimize the impact, and promote smooth recovery, is divided into three categories: vulnerability, adaptability, and conversion ability.

The “Special Act on the Promotion and Support of Urban Regeneration” is the basis of urban regeneration projects [9]. It states that, first, the person possessing the authority to plan the “urban regeneration strategy” has to consider the national urban regeneration basic policy. Second, the “urban rehabilitation revitalization area” can be designated as a strategic target area, to maximize the effectiveness of projects for urban regeneration by concentrating the resources and capabilities of the state and local governments. Third, it discusses “urban regeneration revitalization plans”, which are comprehensively established in urban regeneration areas by linking various urban regeneration projects promoted by the state, local governments, public institutions, and local residents. In other words, it is a system that designates urban regeneration revitalization areas for deteriorated areas and implements urban regeneration revitalization plans for specific targets, after establishing an urban regeneration strategy plan. Therefore, in this study, research is conducted using the “urban rehabilitation plan”, in which the project is actually promoted in urban regeneration areas.

## 3. Methodology

**Figure 1:**
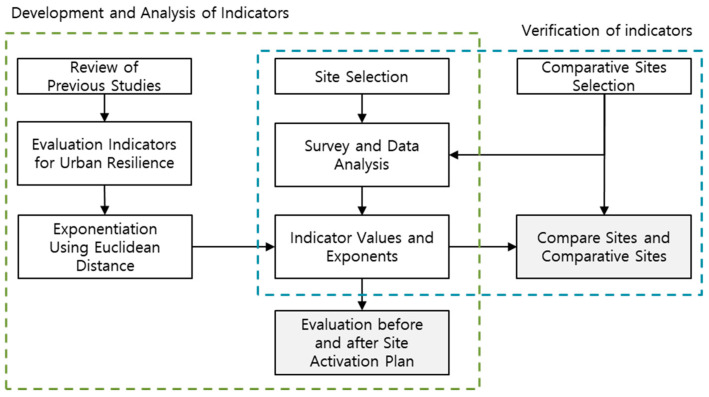
The research process.

The methodology is shown below (Figure 1). First, to establish the measurement method for each indicator and the rating standard for the index value, select an urban resilience evaluation index through a review of previous studies. Second, establish the indexing system using the Euclidean distance based on grading value. Third, after selecting an urban regeneration area for urban resilience evaluation, identify the current status of the target site. Fourth, obtain data on the analysis target site and derive the evaluation index. Finally, check the evaluation index after the regeneration project by reflecting the urban regeneration plan for each target site; then, compare the evaluation index both before and after the project. Additionally, after selecting a non-decadent area near the target site to verify the resilience index, compare and analyze the index values through comparison between regions.

### 3.1. Selection and Classification of Urban Resilience Evaluation Indicators

In previous studies, indicators for evaluating resilience in an urban space have been largely shown as resilience evaluation indicators related to a community perspective [27,28,29,30,31], and climate and disaster [32,33,34,35,36]. Examining the resilience evaluation of studies related to resilience evaluation indicators, the characteristics of resilience (exclusivity, durability, speed, and resource absence) are agreed; however, the components and evaluation units of resilience evaluation are different. To measure urban resilience that requires a diagnosis of the physical environment and social potential, a comprehensive evaluation of the physical infrastructure, natural environment, economy, finance, social and cultural institutions, and the governance policies that constitute the city, must be performed. In this respect, this study attempts to select urban resilience evaluation indicators by comprehensively considering previous studies based on the research indicators of Lee et al. [37] and Choi et al. [6], which set indicators based on physical and natural environmental factors, and socio-economic factors.

**Figure 2:**
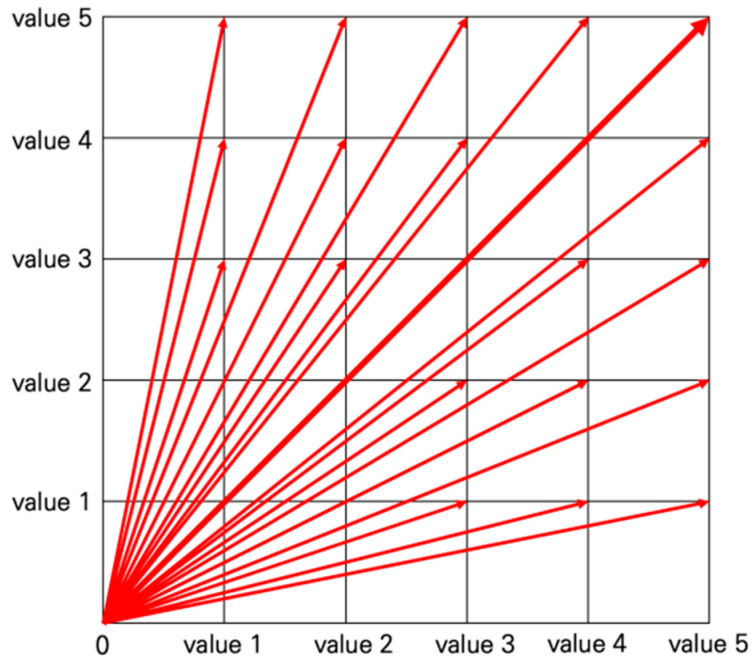
The normalized Euclidean distance.

In this study, the criteria for selecting the urban resilience evaluation index are as follows. First, considering that adapting to external shock and stress further develops a region, safety adaptability and convertibility were set as the representative factors of urban resilience. At this time, the evaluation of the current risk and vulnerability level, and the possibility of short-term changes were considered, rather than the history of disasters in the region. Second, to reflect region-specific factors, natural, social, physical, and administrative characteristics, were considered. Third, urban resilience was set based on data that practitioners can easily collect and evaluate. Fourth, it was intended to develop indicators that could be compared before and after the implementation of urban regeneration projects (the planning and introduction of technologies and facilities), so that the effects and results of urban regeneration projects could be visible. The final 24 indicators were divided into major categories: physical and environmental indicators as the Green Resilient Infrastructure (GRI); and socio-economic indicators as the Interactive Safety System (ISS). Subsequently, they were recategorized by the divisions, including vulnerability, adaptability, and transformability (Figure 2).

The measurement indicators were divided into 12 GRIs, 12 ISSs; a total of 24 items, focusing on the statistical data disclosed and the urban revitalization plan. The measurement of indicators is shown in Table 2. The measured indicators are graded for indexing: the highest grade is 5; the first grade is classified in the order of the lowest; and the reference value setting of the grade is divided based on the following criteria. First, if there is a legal or institutional recommendation, a rating is given based on the corresponding value. Second, if there is no appropriate reference value, it is used as a reference for the national average, minimum, and maximum values. Third, as the declining area is evaluated for resilience, population changes and changes in the number of businesses are evaluated as positive when the rate is maintained.

### 3.2. Results Indexing Using the Euclidean Distance

Each indicator item related to the selected urban resilience comprises independent information, and the unit and size of the data are diverse; thus, indexing is necessary. The weighting method is generally used for indexing but has limitations in validation and human risks; hence, the result may vary depending on the composition of the expert group. Therefore, in this study, the “Euclidian distance value” was calculated as it provides a mathematical relationship between indicators by directly calculating them. This is an indexation method that utilizes the Scala-product of a vector in a geometric space and has been actively used in recent studies, including ecological studies, to mathematically evaluate the relationship between environmental factors.

Urban resilience evaluation is largely performed in three stages—through the process of small → medium → large classification. Small classifications are defined as the fundamental indicators that use intuitive measurements and then composes medium classifications. Subsequently, the urban resilience index is determined by considering two large classifications, GRI and ISS, and each is composed of various medium classifications. In other words, the urban resilience index can be evaluated by changes in the hierarchical spaces of indices in time series. The measurement index values used in this study vary widely in unit and measurement ranges as various temporal sizes and spatial ranges were used. Utilizing the Euclidean distance is considered more efficient as it uses normalized values in which variation is minimized, rather than using the Scala-product of vector, because the spatial range of urban resilience evaluation is enormous. Additionally, urban resilience indexing using normalized Euclidean distance also can deduce the designated spatial index, as shown in Figure 2. Further, it can evaluate the measurements using a matrix of results.

In other words, if the measured value varies from one to five, 25 (5 × 5) classification values can be calculated using two measurements, and it can easily assess the significance as an interpretation of a value. The calculation method of the Euclidean distance for indexing varies, depending on the number of items used for calculation. In terms of urban resilience indicators, the classification can be largely performed using one to three measurements of indicators.

- In the case of one measurement indicator, the height of the Euclidean space is “0”. Thus, use the normalization measurement value.
- In the case of two measurement indicators, use the Euclidean norm of two measurement indicator values in the Euclidean space:

(1) $$∥\alpha ,\beta ∥=\sqrt{\left(\alpha^{2}+\beta^{2}\right)}\;\left(\mathrm{when}\;\alpha \;\mathrm{and}\;\beta \;\mathrm{are}\;\mathrm{two}\;\mathrm{measurement}\;\mathrm{indicators}\right)\;$$

- In the case of three measurement indicators, use the Euclidean norm of three measurement indicator values in the Euclidean space:
  (2) $$∥\alpha ,\beta ,\gamma ∥=\sqrt{\left(\alpha^{2}+\beta^{2}+\gamma^{2}\right)}\;(\mathrm{when}\;\alpha ,\;\beta ,\;\mathrm{and}\;\gamma \;\mathrm{are}\;\mathrm{three}\;\mathrm{measurement}\;\mathrm{indicators})$$
  when raw value was used without normalization, the angle between two measurement indicators was deduced and defined as cos θ. Subsequently, calculate to deduce the Scala-product of a vector multiplied by the Euclidean norm. To deduce the index from the calculated Euclidean distance, two steps are required: (1) the normalization of the Euclidean distance; and (2) translation to the selected range. For the normalization of the distance, Min–Max Normalization is used to interpret the meaning of the value measurement, as the calculated index can vary widely, depending on the number of indices and value range:
  (3) $$\left(\alpha -A_{min}\right)/\left(A_{max}-A_{min}\right)$$

- α is the measurement indicator value, *A_max_* is the maximum value of measurement indicator, and *A_min_* is the minimum value of measurement indicator.

The Min–Max Normalization normally presents its result as a value between 0 and 1, but the range was expanded to 1–100 or 50–100 to better interpret the meaning of the value:

(4) $$\left(B-A\right)\alpha +A$$

**Figure 3:**
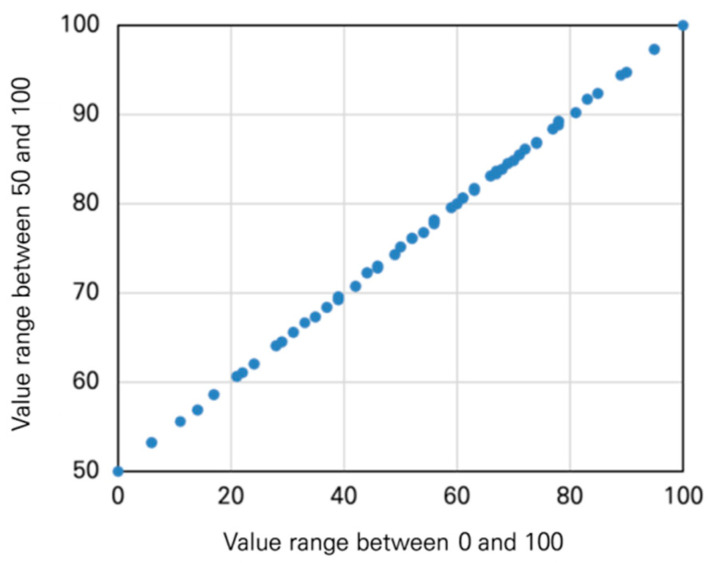
The correlation between two different value ranges.

- α is the measurement indicator value, Β is the maximum value within the range, and A is the minimum value within the range.
- Note: the range of the values does not impact the magnitude and position of the basis of value (Figure 3).

In this study, the formula of the urban resilience index using Euclidian distance is as follows:

(5) $$I_{s}=\sqrt{\sum Site_{ij}^{2}}$$

𝑠 is the set of {GRI-Vulnerability, GRI-Adaptability … ISS-Transformability}, and 𝑖 is the set of sites, $Site_{ij}$ is the *j*-th indicator of the $Site_{i}$.

### 3.3. Target Sites

Baeknyeon village in Indong-chon (Site-A), an open port culture street in Mokpo (Site-B), and Yangyoujeong village in Seosan (Site-C), were designated as target areas for urban resilience evaluation. Based on an on-site survey of the target sites, Site-A is an area with few open spaces, many narrow alleys, and high disaster damage (grade 1), and is vulnerable to disasters such as heatwaves and fires. In the case of Site-B, houses at risk of collapse, such as empty houses and abandoned houses on hills, accounted for more than 10% of total houses, suggesting safety problems. Site-C is in a relatively better condition compared to the other two target sites and has a relatively low vacancy rate of 0.5%. However, it is vulnerable to fire because of narrow alleys; therefore, the urban regeneration project is underway focusing on this point (see Table 3).

To verify the urban resilience index, nonurban regeneration zones near the three target sites were selected as comparative groups (CA, CB, and CC). Comparative group areas are in the same administrative district and have a similar size, with better conditions of physical environment than the target areas. This is because an area in good condition is needed to compare the resilience index. Through comparison with nearby target sites, we attempted to verify whether the index value could represent the state of each target site.

### 3.4. Data

**Figure 4:**
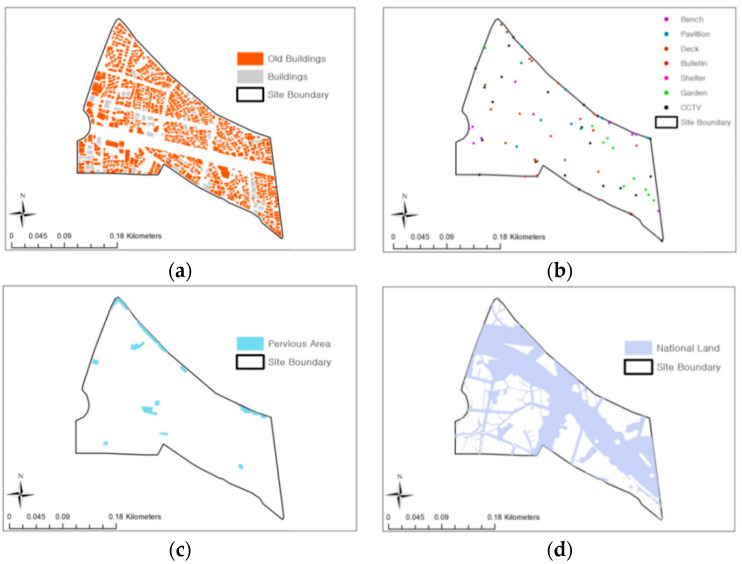
The examples of data construction and analysis of Site-A: (**a**) GA-3; (**b**) IT-4; (**c**) GA-2; and (**d**) GT-2.

To derive the index values of urban regeneration areas, data values for three target sites were first constructed according to the measurement method shown in Table 2 (see Figure 4).

The data were collected before the urban regeneration plan (2017~18), and the spatial range is the target site or city, county, and district, as those are the minimum units where data can be collected. However, among the indicators, field survey indicators for IA-1 and IA-4 could not collect previous data surveys; hence, the survey value at the current time was applied. For postproject data, current data have to be utilized; however, the most recent public statistics are from 2020. Thus, it has limitations in terms of reflecting the holistic impact of the urban revitalization project. Therefore, postproject values were deduced by considering the urban regeneration revitalization plan, which is a plan to promote the project.

## 4. Results

The results of grading based on the established target site index value are shown in Table 4.

Examining the preproject grade and values, GA-1, GA-5, GT-1, and GT-4 are extremely low in GRI, as they show the characteristics of declining areas. This reveals the reality of the lack of leisure and community space because of a high concentration of houses and the lack of parking spaces, which are the characteristics of old cities. Further, it was confirmed that there were no facilities to evacuate/respond to a disaster. The most characteristic feature of ISS is population decline (IV-2) due to urban decline. Additionally, IV-3 and IV-4—indicators representing the socially and economically vulnerable class—were low at 1 to 2 grades. This shows the socio-economic decline of the region due to population decline, which is a typical phenomenon of local decline in Korea.

Examining the characteristics of urban resilience indicators before and after urban revitalization projects by target sites, only a few indicators identified the change. This is because the urban regeneration project is not a project to develop the entire target site. In the case of Site-A, GA-2 and GT-1 indicators rose significantly to Grade 1 → Grade 5, and IA-1 and IT-4 indicators also rose. For Site-B, the indicators of GA-1, GA-2, GT-1, and IA-1 improved, and Site-C showed improved ratings in GA-1, GT-1, IA-1, and IT-4. Further, GT-1 and IA-1 showed improvement in ratings in all three target sites, while GA-1, GA-2, and IT-4 indicators showed an increase in ratings in two or more target sites. This can be viewed as a result of the current characteristics of urban revitalization projects, such as securing open spaces, green spaces, rest areas, and installing CCTV.

After calculating the grade based on the index value, the index values for each target site were derived using the Euclidean distance. The results are as follows (see Table 5). First, among the three target sites, the index value was the highest with 63 points, followed by Site-A, 48 points, and Site-B, 44 points. Second, as a result of deriving the urban resilience index value after the urban regeneration project plan, Site-A rose 10 points (48 → 58 points), Site-B rose nine points (44 → 53 points), and Site-C rose seven points (63 → 70 points). When the urban regeneration project plan is applied to all three target sites, the value of the urban resilience index increases.

To check whether the values of the urban resilience index are relatively low in the declining areas, an analysis of the three destinations and comparison groups was conducted. The results showed 58 points for CA, higher than that of Site-A (48 points), 57 points for CB, higher than that of Site-B (44 points), and 67 points for CC, higher than that of Site-C (63 points).

## 5. Conclusions

Selecting urban resilience indicators through a review of previous studies, and applying them to the target sites, showed the current status of urban regeneration areas, or declining areas, in Korea. The indicators corresponding to GRI (GA-1, GA-5, GT-1, and GT-4) show that the grades are low because of an inferior environment and the lack of infrastructure in aging cities. The social and economic decline due to population decline is shown in the grades of ISS (IV-2, IV-3, and IV-4). All three target sites are urban regeneration project areas, and these grades reflect this fact. Comparing the indexation scores, it was shown in the order of Site-B < Site-A < Site-C, and it is judged that the perception during the actual status survey is like this. However, it is difficult to judge that areas with high scores must have higher urban resilience than areas with low scores. This is because the index value was derived by reflecting the characteristics of each city, county, and district. Nevertheless, the increase in the index value before and after the urban regeneration plan implies an improvement in urban resilience in the region; it is judged that comparisons in the same city, county, and district will be valid. The reason for this is that the same statistical data, including disaster damage and population composition, are used, and they are administratively and geographically located in the same location. Reflecting this, the index values (58, 57, and 67 points) were higher than those of the target sites (48, 44, and 63 points) in all three comparative areas located adjacent to the target sites.

Urban regeneration projects in Korea have thus far focused on the participation of residents, the promotion of communication, and securing space to improve it; the consideration of disasters has been insufficient. In particular, it is difficult to improve the physical environment dramatically because of various factors such as the aging of the residential environment, the concentration of socially vulnerable groups, and budget limitations. However, urban resilience considers the physical environment, and reflects the social and economic environment. In other words, urban regeneration areas with limitations in improving the physical environment should endeavor to improve responses to emergency situations, and damage recovery ability, by securing local response procedures and delivery systems, public safety management personnel, volunteer workers, and community organizations. Moreover, the systematic management of vacant and old houses found in declining areas is necessary for improving the physical environment. The increase in unmanaged vacant houses accelerates regional decline, leading to safety issues. This is why Kim and Nam [19] emphasized the management of vacant houses and suggested a comprehensive system for budget expansion, vacant house investigation, and DB management. In the case of old houses, if full repairs are difficult then improving the residential environment in preparation for disasters, such as renovating the roof and installing fire extinguishing facilities, is necessary.

Globally, including in Korea, natural disasters caused by climate change are becoming more severe. In particular, 2022 would be the year when the world realized the gravity of climate change as a result of record heavy rain, heatwaves, and drought. Going forward, the response to climate change and disaster will be the biggest issue in urban planning. The significance of disaster response will grow, and urban regeneration projects should consider this. It is impossible to nullify the impact of disasters completely. Therefore, it is necessary to recover from them and create a more developed counter-disaster structure for the future. To this end, the urban resilience index of this study can be used for urban resilience evaluation. Urban resilience can ultimately facilitate the overall stability of a region. Although it is difficult to use the urban resilience index in this study to compare different regions, it can be used for within-region time-series comparison. The maximum score of the index is 100 points, but it is difficult to define an appropriate resilience score. Thus, to increase the overall resilience of a region, efforts should be made to raise the overall score of each region. Urban regeneration plans, and urban planning, should be established based on urban resilience.

The purpose of this study was to select a practical evaluation index that can assess urban resilience and apply it to evaluate different target sites’ resilience, to deduce the direction of the urban regeneration plan. To this end, indicators were set based on data accessibility to the urban regeneration practitioners and indexed using Euclidean distance. Further, we compared actual urban regeneration sites in nearby areas by applying indicators and confirmed that the indicators and index could be used. However, to increase the efficiency and practicality of the indicators and index, it is recommended to compare only the target sites within the same city, county, and district. Moreover, it is regrettable that it is difficult to compare them with other regions. In future studies, through detailed studies by measurement indicators, appropriate resilience indicators and index standards should be prepared to respond flexibly to external shocks. Additionally, as this study analyzed changes in the physical indicators of urban regeneration plans, because of the limitations of data subscription, more comprehensive resiliency-indicator studies should be conducted, which include improvements in social indicators.

## Figures and Tables

**Table 1:** The keywords of “urban resilience” definition.

Organizations	RM	SS	AR	SAM	RE	GP	SA
OECD	-	●	●	●	-	-	●
UN-HABITAT	-	●	-	●	●	-	-
International Council for Local Environmental	-	●	●	●	●	-	-
United Nations Office for Disaster Risk Reduction	●	-	●	-	●	-	-
Rockefeller Foundation	-	●	-	●	-	●	-
World Bank	-	●	-	●	●	-	-
USAID	●	●	-	●	●	●	-
100 Resilient Cities	-	●	-	●	-	●	-
Resilient Europe	-	●	-	-	●	●	-
Global Alliance for Resilience	-	●	●	●	●	-	●

Note. RM: risk management; SS: shock stress; AR: absorption and reaction; SAM: survival adaptation and maintenance; RE: recovery; GP: growth and prosperity; SA: sustainability; ●: Show if the definition is included; Source: based on [6,23].

**Table 2:** The urban resilience evaluation indicators.

Large	Medium	Small	Indicators			Unit
GRI	Vulnerability	Disaster damage	GV-1	Property damage from disaster	Total amount of property damage due to disasters in last 10 years	₩ / m^2^
			GV-2	Human life damage from disaster	Total casualties due to disasters in last 10 years	No. of people / m^2^
		Size of vulnerable area	GV-3	Special purpose district	Combined area of the district	m^2^
	Adaptability	Ecological adaptability	GA-1	Open space	Percentage of open space area in urban planning facilities	%
			GA-2	Green parks and infrastructure	Proportion of green area and other green spaces	%
		Safety of buildings and structures	GA-3	Aged building	Percentage of 20 years + old buildings	%
			GA-4	Building density	Ratio of floor area of buildings in the target site	%
			GA-5	Structural stability	Percentage of wooden or masonry structured buildings	%
	Transformability	Scalability of community facilities	GT-1	Community facilities accessibility	Number of schools + public health centers + administrative facilities + parks	No. of facilities/m^2^
			GT-2	Land ownership status	Ratio of publicly owned land	%
		Adequacy of response infrastructure	GT-3	Road accessibility	Ratio of buildings adjacent to roads with a width of 4 m or more	%
			GT-4	Accessibility to evacuation facilities	Number of civil defense evacuation facilities	No. of facilities/m^2^
ISS	Vulnerability	Population composition	IV-1	Vulnerable population	Proportion of vulnerable population (age less than 14 and more than 65)	%
			IV-2	Population change	Population change rate of a region in last 10 years	%
		Society and economy	IV-3	Economically vulnerable class	Proportion of basic livelihood recipient + single-parent family beneficiary + foreign residents	%
			IV-4	Small business owners	Percentage of small business owners	%
	Adaptability	Pre-emptive response system	IA-1	Customized alarm system	Number of alarm systems and notification systems + CCTV	No. of systems/ 10,000 m^2^
			IA-2	Vacant house maintenance project	Percentage of vacant houses	%
		Tailored (Emergency) support system	IA-3	Emergency medical (protection) system	Area that can be opened for emergency medical support	m^2^/person
			IA-4	Public safety management personnel	Local government safety management (police officers + firefighters + public officials dedicated to social welfare) personnel	No. of personnel/1,000 people
	Transformability	Availability of human resource	IT-1	Disaster management budget	Average value of local government disaster management funds raised in last five years	₩1M/1,000people
			IT-2	Volunteer	Number of local government registered volunteers	No. of volunteers/ 1,000 people
		Risk communication activity	IT-3	Resident/Business/Socio-economic organization	Percentage of participants in the organization (such as residents, merchants, etc.) + subscribers to community mapping services	%
			IT-4	Community activity spaces	Number of outdoor community places (chairs, tables, etc.)	No. of places/ 10,000 m^2^

**Table 3:** The condition of target sites.

Sites	Site A	Site B	Site C
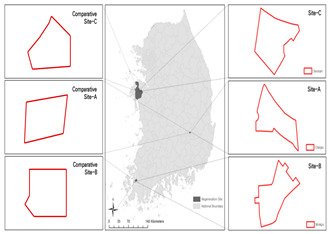	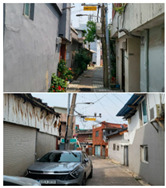	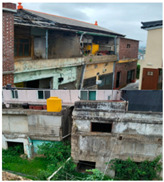	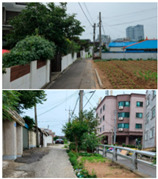
	Area: 174,452 m^2^ Project period: 2019~2022	Area: 294,831 m^2^ Project period: 2017~2022	Area: 109,000 m^2^ Project period: 2020~2023
	Comparative Site A	Comparative Site B	Comparative Site C
	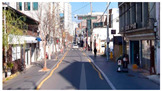	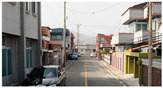	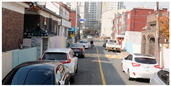
	Area: 246,526 m^2^	Area: 597,472 m^2^	Area: 190,141 m^2^

**Table 4:** The value and grade by target site.

Category	Site-A				Site-B				Site-C			
	Preproject		Postproject		Preproject		Postproject		Preproject		Postproject	
	Value	Grade	Value	Grade	Value	Grade	Value	Grade	Value	Grade	Value	Grade
GV-1	649.29	1	649.29	1	241.82	4	241.82	4	82.56	5	82.56	5
GV-2	7.48	1	7.48	1	1.95	3	1.95	3	0.29	5	0.29	5
GV-3	3.70	5	3.70	5	5.81	4	5.81	4	2.68	5	2.68	5
GA-1	0.00	1	0.79	1	0.00	1	3.10	3	0.00	1	4.04	3
GA-2	0.00	1	5.47	5	2.94	3	10.21	5	4.71	5	16.03	5
GA-3	87.55	2	87.55	2	80.23	2	80.23	2	89.17	2	89.17	2
GA-4	52.45	3	52.45	3	31.12	4	31.12	4	26.95	5	26.95	5
GA-5	22.49	1	22.49	1	40.82	1	40.82	1	13.38	1	13.38	1
GT-1	0.00	1	1.20	5	0.47	2	1.19	5	0.24	1	2.57	5
GT-2	30.50	3	30.50	3	32.21	3	32.21	3	19.14	1	19.14	1
GT-3	50.31	5	50.31	5	41.91	5	41.91	5	55.59	5	55.59	5
GT-4	0.00	1	0.00	1	0.00	1	0.00	1	0.00	1	0.00	1
IV-1	27.30	3	27.30	3	25.92	3	25.92	3	58.64	1	58.64	1
IV-2	−23.81	1	−23.81	1	−25.47	1	−25.47	1	1.46	5	1.46	5
IV-3	10.00	1	10.00	1	8.98	2	8.98	2	4.66	2	4.66	2
IV-4	54.90	1	54.90	1	59.58	1	59.58	1	50.32	1	50.32	1
IA-1	1.49	2	2.69	3	0.88	1	2.17	3	2.48	3	5.96	5
IA-2	6.90	3	6.90	3	11.98	1	11.98	1	0.51	5	0.51	5
IA-3	0.68	1	0.68	1	1.77	2	1.77	2	2.38	3	2.38	3
IA-4	4.83	3	4.83	3	4.19	3	4.19	3	3.25	2	3.25	2
IT-1	69.31	5	69.31	5	40.00	3	40.00	3	40.27	3	40.27	3
IT-2	245.66	1	245.66	1	196.34	1	196.34	1	266.49	2	266.49	2
IT-3	11.51	3	11.51	3	8.71	2	8.71	2	8.35	2	8.35	2
IT-4	2.58	4	3.10	5	0.78	1	1.15	1	2.57	4	3.12	5

**Table 5:** The comparison of urban resilience index.

Before Project	After Project	Comparative Group
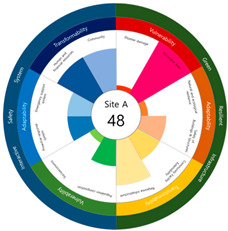	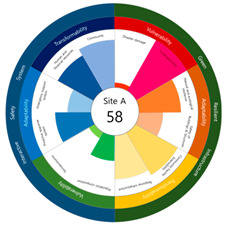	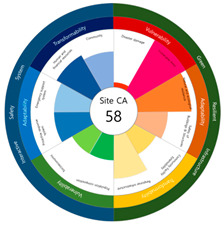
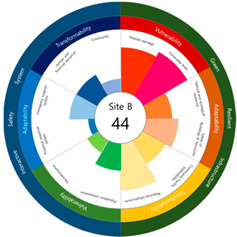	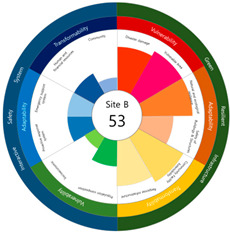	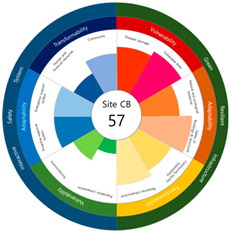
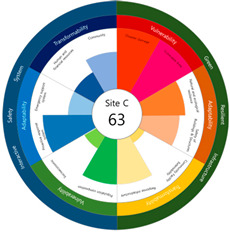	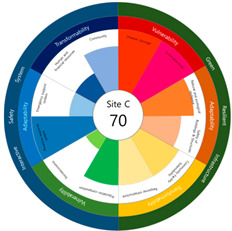	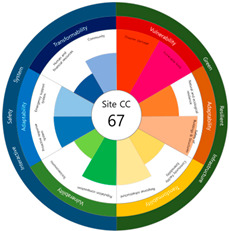

## Data Availability

Not applicable.

## References

[1] Park S. (2015). Enhancing Regional Resilience in Response to Crisis. Land.

[2] Jung H., Yang C. (2018). Research trend of resilience studies in public administration and public policy using keywords network analysis. Korean J. Policy Anal. Eval..

[3] Marchese D., Reynolds E., Bates M.E., Morgan H., Clark S.S., Linkov I. (2018). Resilience and sustainability: Similarities and differences in environmental management applications. Sci. Total Environ..

[4] Sanchez A.X., Van der Heijden J., Osmond P. (2018). The city politics of an urban age: Urban resilience conceptualisations and policies. Palgrave Commun..

[5] Lee G., Jin D., Song S., Choi H. (2019). Text analysis on the research trend of ‘Resilience’ in Korea: Focus on climate change and urban disaster. J. Clim. Chang. Res..

[6] Choi H., Lee G., Eo S. (2021). Development of urban resilience evaluation indicators and policy application. KEI Environment Forum.

[7] Shin Y., Lee S., Chang K., Yang D. (2021). Development of comprehensive diagnosis model for urban space in deteriorated areas: Focusing on disaster risk and resiliency. J. Korea Plan. Assoc..

[8] Jeon E., Byun B. (2017). A study on the Development and application of community resilience evaluation indicators for responding to climate change. Geogr. J. Korea.

[9] Korean Law Information Center Special Act on the Promotion and Support of Urban Regeneration. https://www.law.go.kr/.

[10] Han J., Nam J. (2020). A study on the effects of the decline of industrial complex on the urban areas. J. Korean Reg. Dev. Assoc..

[11] Kwon O., Kang E., Ma K. (2019). A study on the impact of the urban decline on the subjective well-being of residents. J. Korean Reg. Sci. Assoc..

[12] Jung E. (2017). A study about the planning housing cluster for sustainable community of declining regions in non-urban area. J. Korean Reg. Dev. Assoc..

[13] Adger W.N. (2010). Social capital, collective action and adaptation to climate change. Econ. Geogr..

[14] Kwon Y., Lee J., Kim H., Oh J. (2013). Comprehensive urban regeneration design strategy of abandoned railroad area through citizen-participation: Focused on the case of Gwangyang-si abandoned railroad park. J. Urban Des. Inst. Korea.

[15] Yoon Y. (2014). The approvement and review of maeulmandeulgi through resident’s participation for community oriented urban renewal development. SH Urban Res. Insight.

[16] Lee N., Ahn J. (2017). ‘General type of neighborhood regeneration’ urban regeneration project and promotion of resident-participation: Focusing on Garibong-dong, Guro-gu, Seoul. J. Korean Urban Geogr. Soc..

[17] Lee J. (2020). A case study on residents’ participation in urban regeneration project. SH Urban Res. Insight.

[18] Kang J., Moon K. (2022). The effect of local social properties on residents’ intention to participate in urban regeneration projects. J. Korean Hous. Assoc..

[19] Kim J., Nam J. (2016). A study on vacant house distribution and management of urban declining area. J. Korean Reg. Sci. Assoc..

[20] Im H., Ahn M., Lee C., Lee S., Lee J. (2021). Development of a comprehensive diagnosis index for disasters in declining areas and comparison of risks between regions: A case of Seoul. J. Korean Reg. Sci. Assoc..

[21] Kim Y., Park B., Jung H. (2018). Climate change vulnerability of roads to heavy snow: Establishing new assessment criteria on VESTAP. Korean J. Urban Stud..

[22] Kim J., Park J., Cho B., Lee S. (2020). A comparative analysis of disaster vulnerability factors between declining areas and urban areas. J. Digit. Contents Soc..

[23] OECD (2018). Indicators for Resilient Cities.

[24] Walker B., Honning C.S., Carpenter S.R., Kinzig A. (2004). Resilience, adaptability and transformability insocial-ecological systems. Ecol. Soc..

[25] Folke C., Carpenter S.R., Walker B., Scheffer M., Chapin T., Rockstrom J. (2010). Resilience thinking: Integrating resilience, adaptability and transformability. Ecol. Soc..

[26] Kim J., Im J., Lee S. (2018). Development and applicability of resilient city criteria for adopting resilient city model: Focused on Hamburg water cycle jenfelder au, Germany. J. Urban Des. Inst. Korea.

[27] Cutter S.L., Ash K.D., Emirich C.T. (2014). The geographies of community disaster resilience. Glob. Environ. Chang..

[28] U.S. IOTWS (2007). How Resilient Is Your Coastal Community? A Guide for Evaluating Coastal Community Resilience to Tsunamis and Other Hazards.

[29] Kang S., Cho S., Hong S. (2013). A Policy Implication for Community Resilience from Natural Disasters.

[30] Yoon D., Kang J., Brody S. (2016). A measurement of community disaster resilience in Korea. J. Environ. Plan. Manag..

[31] Sherrieb K., Norris F.H., Galea S. (2010). Measuring capacities for community resilience. Soc. Indic. Res..

[32] EPA (2017). Development of a Climate Resilience Screening Index (CSRI): An Assessment of Resilience to Acute Meteorological Events and Selected Natural Hazards.

[33] Miola A., Paccagnan V., Padadimitriou E., Mandrici A. (2015). Climate Resilient Development Index: Theoretical Framework, Selection Criteria and Fit-for-Purpose Indicators.

[34] Kim D., Song S., Kang S., Kwon T., Kim J., Nam K., Yoon D., Lee D., Jung J., Jo S. (2016). Urban Climate Resilience: Operationalization and Evolution (II).

[35] Parsons M., Morley P. (2017). The Australian natural disaster resilience index. Aust. J. Emerg. Manag..

[36] Cutter S.L., Burton C.G., Emrich C.T. (2010). Disaster resilience indicators for benchmarking baseline conditions. J. Homel. Secur. Emerg. Manag..

[37] Lee G., Jung Y., Eo S., Choi H. (2021). A primary study to develop an evaluation index for urban resilience in declining areas: Utilization of policy network analysis and fuzzy multiple-criteria decision-making method. J. Clim. Chang. Res..

